# Analysis of Notch Signaling-Dependent Gene Expression in Developing Airways Reveals Diversity of Clara Cells

**DOI:** 10.1371/journal.pone.0088848

**Published:** 2014-02-21

**Authors:** Arjun Guha, Michelle Vasconcelos, Rui Zhao, Adam C. Gower, Jayaraj Rajagopal, Wellington V. Cardoso

**Affiliations:** 1 Pulmonary Center, Boston University School of Medicine, Boston, Massachusetts, United States of America; 2 Center for Regenerative Medicine, Massachusetts General Hospital, Boston, Massachusetts, United States of America; 3 Pulmonary and Critical Care Unit, Departments of Internal Medicine and Pediatrics, Massachusetts General Hospital, Boston, Massachusetts, United States of America; 4 Boston University Clinical and Translational Science Institute, Boston, Massachusetts, United States of America; Children’s Hospital Los Angeles, United States of America

## Abstract

Clara cells (CCs) are a morphologically and operationally heterogeneous population of Secretoglobin Scgb1a1-expressing secretory cells that are crucial for airway homeostasis and post-injury repair. Analysis of the extent and origin of CC diversity are limited by knowledge of genes expressed in these cells and their precursors. To identify novel putative markers of CCs and explore the origins of CC diversity, we characterized global changes in gene expression in embryonic lungs in which CCs do not form due to conditional disruption of Notch signaling (Rbpjk^CNULL^). Microarray profiling, Real Time PCR (qRT-PCR), and RNA *in situ* hybridization (ISH) identified eleven genes downregulated in the E18.5 airways of Rbpjk^cnull^ compared to controls, nearly half not previously known to mark CCs. ISH revealed that several genes had overlapping but distinct domains of expression of in the normal developing lung (E18.5). Notably, *Reg3g*, *Chad*, *Gabrp* and *Lrrc26* were enriched in proximal airways, *Hp* in the distal airways and *Upk3a* in clusters of cells surrounding Neuroepithelial Bodies (NEBs). Seven of the eleven genes, including *Reg3g*, *Hp*, and *Upk3a*, were expressed in the adult lung in CCs in a pattern similar to that observed in the developing airways. qRT-PCR-based analysis of gene expression of CCs isolated from different airway regions of B1-EGFP reporter mice corroborated the spatial enrichment in gene expression observed by ISH. Our study identifies candidate markers for CC-precursors and CCs and supports the idea that the diversification of the CC phenotype occurs already during embryonic development.

## Introduction

Secretory cells are among the most abundant cell type in the mammalian airways. These cells produce a variety of secretory products, including mucus, that are key regulators of the airway inflammation and homeostasis [1]. Secretory cells are also known to be heterogeneous in their morphology, molecular composition and responses to environmental challenge. This heterogeneity is observed across species and correlates with position of cells along the proximal-distal (P-D) axis of the airway epithelium [1], [2]. Secretoglobin Scgb1a1, the most extensively characterized marker for airway secretory cells, labels a subpopulation of secretory cells in distal airways of the human lung (also called Clara cells, CCs). Scgb1a1 is also expressed in secretory cells in the mouse lung but, unlike in humans, Scgb1a1^+^ secretory cells in mice (hereafter referred to as CCs) are the predominant secretory cell type in this species. In this study, we focus on the development and diversification of CCs in the mouse lung.

Several studies have demonstrated differences among CCs in the murine airways. In response to acute or chronic inflammatory stimuli, only CCs in the proximal airways undergo a rapid transformation to mucus-producing goblet cells [3], [4]. The transcription factor SP-DEF (SAM pointed domain-containing Ets transcription factor) has been shown to have an essential role in the conversion of CCs to goblet cells. Importantly, when SP-DEF was expressed in all CCs under the control of Scgb1a1 promoter, it induced rapid transformation of only proximal CCs into goblet cells [4]. This demonstrates intrinsic differences in proximal and distal airway CCs. Apart from a role in airway inflammation; CCs serve as airway progenitors that contribute to homeostasis and post-injury repair. Studies on lung injury-repair have also indicated that CCs are a genetically heterogeneous population. Lineage-tracing experiments that have examined the contribution of CCs to airway renewal during homeostasis show that tracheal CCs are like transit-amplifying cells that do not self-renew extensively, whereas bronchial and bronchiolar CCs are long-term progenitors that both self-renew and generate ciliated cells [5]. Certain CCs associated with Neuroepithelial Bodies (NEBs) and located near Bronchioalveolar Duct Junctions (BADJs) invariably survive Naphthalene-induced CC ablation (Nap) [2], [6], [7].

It has been proposed that the observed differences among CCs in the adult lung have a developmental basis [8]. A study that examined the distribution of different members of the Secretoglobin family (Scgb1a1, Scgb3a2, and Scgb1a1) found that these gene products have distinct spatial distributions in the adult lung. While the expression of the Secretoglobins Scgb1a1 and Scgb3a2 was widespread, expression of Scgb3a1 was enriched in the proximal airways. Importantly, these differences in the expression patterns of the three Secretoglobins were evident from late stages in embryonic development [8].

Notch signaling has an essential role in specification of CCs during lung development [9], [10], [11], [12]. Lineage analysis has shown that airway progenitors that activate Notch signaling during development adopt a CC fate [10]. Sonic Hedgehog (*Shh*)-cre dependent ablation of Rbpjk, the transcription factor essential for canonical Notch signaling (hereafter Rbpjk^CNULL^) lack CCs and have supernumerary ciliated and neuroendocrine cells [9], [10].

In spite of the overwhelming evidence for functional heterogeneity of CCs, little is known about the molecular markers that distinguish CC subpopulations and the origins of this diversity. In this study, we utilize Rbpjk^CNULL^ lungs from embryos at E18.5 to screen for novel genes expressed in differentiating CCs. Since CCs are not specified in Rbpjk^CNULL^ and are the only cell type affected in this manner, we reasoned that genes expressed in differentiating CCs would be downregulated in these mutant lungs and expressed in adult CCs. Using a combination of microarray profiling, quantitative RT-PCR and ISH we found a number of genes not previously known to label CCs. Our study sheds light on the spatiotemporal regulation of CC differentiation and diversification in the developing lung and identifies potential markers for distinct subpopulations of CCs along the P-D axis of the respiratory tract.

## Results

### Transcriptional Profiling of E18.5 Lungs from Notch-deficient Rbpjk^CNULL^ Mice Identifies Genes Enriched in CCs

**Figure 1:**
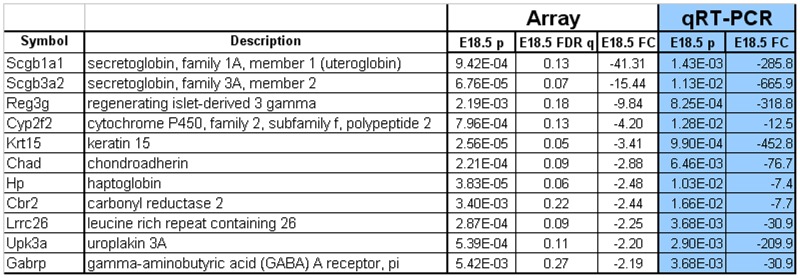
Microarray-based identification of mRNAs downregulated in Rbpjk^CNULL^ lungs at E18.5 and validation using qRT-PCR. Genes downregulated greater than two-fold (p-value <0.05 and FDR-q value <0.28) are shown here. FC = fold change; FDR-q = Benjamini-Hochberg False Discovery Rate. A total of n = 3 lungs per condition from control (CTRL) and *Shh* ^cre/+^; Rbp ^Flox/Flox^ (Rbpjk^CNULL^) were used for the respective analyses.

There is currently limited knowledge of the markers that can be used to investigate the program of differentiation and diversification of the airway epithelium in the developing trachea and lung. This is particularly true for CCs, the most abundant and functionally diverse secretory cells of the murine lung. Cells expressing Scgb1a1, the definitive marker for CCs, can be detected throughout the developing airways at E18.5. At this stage, although already expressing Scgb1a1, CCs are still developing and are known to maturate only postnatally [5], [13]. Developing airways devoid of Notch signaling (Rbpjk^CNULL^) lack all Scgb1a1^+^ cells at this stage [9], [10]. Thus, to identify novel genes expressed in CC-precursors and CCs we compared the transcriptional profiles of lungs from control and Notch-signaling deficient Rbpjk^CNULL^ mice at E18.5. Of the 21,225 unique mouse Entrez Gene IDs represented on the Affymetrix Mouse Gene 1.0 ST v1 arrays, eleven gene products were downregulated greater than two-fold in Rbpjk^CNULL^ lungs (p<0.05, Benjamini-Hochberg False Discovery Rate q<0.28). These genes were selected for further analysis as candidate markers for CC-precursors and CCs (Fig. 1). Quantitative real-time PCR (qRT-PCR)-based analysis of the expression of these eleven genes in lung homogenates from E18.5 confirmed that the differences observed by transcriptional profiling were indeed significant (n = 3 lungs per condition, Fig. 1).

**Figure 2:**
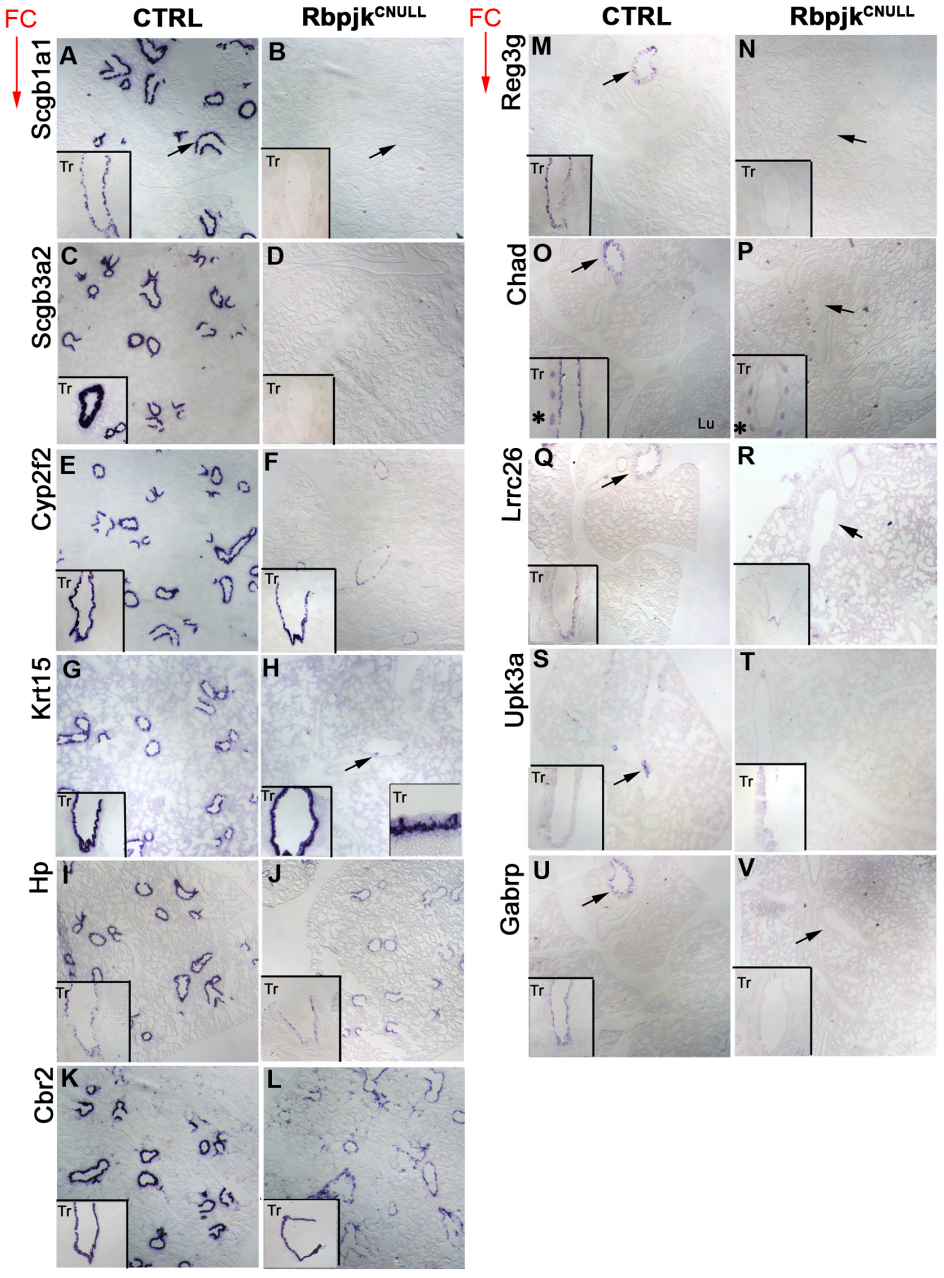
Expression patterns of the genes identified by transcriptional profiling. RNA *in situ* hybridization (ISH) analysis of the expression of candidate genes in in control and Rbpjk^CNULL^ lungs at E18.5. All genes were expressed in the airways in widespread (**A–K**) and localized (**M–U**) patterns and downregulated in the Rbpjk^CNULL^ lungs (**B–L**, **N–V**). *Chad* expression was detected in precursors of the cartilage (**O**, asterisk) and this expression was unaffected in Rbpjk^CNULL^ (**P**, asterisk). Arrows in the panels indicate comparable regions of the airways. Red arrows indicate that the panels have been vertically ordered with respect to Fold Change (FC) in gene expression reported by microarray profiling. ISH on CTRL and Rbpjk^CNULL^ sections was performed simultaneously and the staining was developed for the same period for time. Tr = Trachea, Lu = Lung.

Next we investigated the spatial distribution of these genes by ISH analysis of E18.5 control lungs. We found the expression of all eleven genes largely restricted to the airway epithelium (Fig. 2), except *Chad* (Chondroadherin), which was also present in precursors of the cartilage in the trachea and proximal airways (Fig. 2O–P, insets). ISH analysis of Rbpjk^CNULL^ showed that all eleven were downregulated in these lungs (Fig. 2). We noted that the expression of *Chad* in cartilage precursors was unaffected in Rbpjk^CNULL^ (Fig. 2P inset). Expression of seven of these eleven genes (*Scgb1a1, Scgb3a2*, *Reg3g*, *Chad, Lrrc26, Upk3a*, *Gabrp)* was abolished in the airway epithelium of E18.5 Rbpjk^CNULL^, suggesting that these genes likely label CCs or their precursors present in the trachea or lung at E18.5. The other four genes showed either an overall reduction (*Hp, Cbr2*) or a regional (*Cyp2f2*, *Krt15)* reduction in the ISH signals, suggesting that these genes were also expressed in other populations of cells, presumably precursors of non-secretory cells. Indeed double ISH/immunohistochemistry (IHC) of E18.5 lungs revealed colocalization of some of these genes (*Cbr2*, *Hp*, *Krt15*) with markers of ciliated or basal cells (data not shown).

The most striking finding of the ISH analysis at E18.5 was the identification of two general patterns of gene expression. One group of genes, represented by *Scgb1a1, Scgb3a2, Cyp2f2*, *Krt15, Hp, and Cbr2,* showed a broad domain of mRNA expression extending from the trachea to the terminal bronchiole (Fig. 2.G–L). Subtle differences in proximal-distal (P–D) transcript distribution are expected since the proportion of CCs in the distal airways is known to be higher. The density of Scgb1a1 staining in the proximal airways is less than in the distal airways (compare Fig. 2A (inset) and Fig. 2A). Among the other genes that were broadly expressed, *Cyp2f2, Krt15*, and *Cbr2* transcripts were evenly expressed throughout the epithelium, *Scgb3a2* was expressed at higher levels in the proximal airways (Fig. 2C, inset) and *Hp* was expressed at lower levels in proximal airways (Fig. 2I, inset). Two-color fluorescence- ISH for *Scgb1a1* and *Scgb3a2* indicated that some *Scgb3a2*-expressing cells in the proximal airways expressed low to negligible levels of *Scgb1a1* at this stage (not shown).

The second group of genes, represented by *Reg3g*, *Chad*, *Lrrc26*, *Upk3a*, and *Gabrp,* showed restricted regional or local expression patterns (Fig. 2M–V). Among these *Reg3g*, *Chad, Gabrp* and, *Lrrc26* transcripts were detected in proximal epithelial cells in the trachea and extrapulmonary airways, but not in the intrapulmonary airways. *Upk3a* expression was observed in highly localized clusters of cells in the intrapulmonary airways (Fig. 2S, T). We have shown previously that these clusters of Upk3a-expression are associated with presumptive Neuroepithelial Bodies (NEBs) in the intrapulmonary airways [12].

The distinct distribution of these genes in E18.5 controls and their downregulation in Rbpjk^CNULL^ strongly suggested that at this stage they label subpopulations of CCs or precursors.

### Several Genes Downregulated in Rbpjk^CNULL^ at E18.5 Mark CCs in the Adult Lung

**Figure 3:**
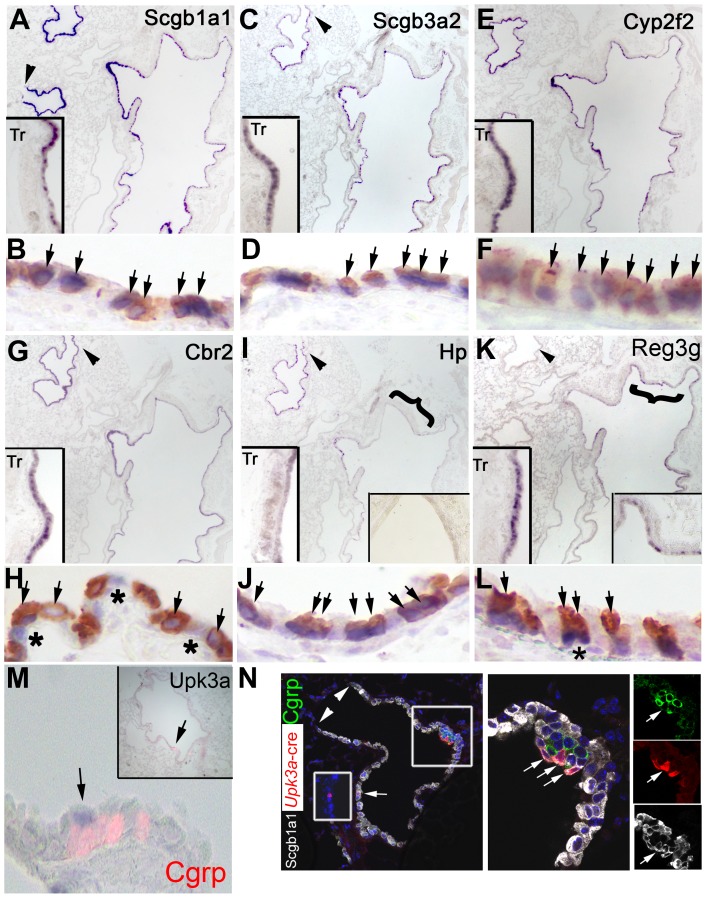
Several genes downregulated in Rbpjk^CNULL^ at E18.5 are expressed in adult CCs. **(A–H)** Transcripts that were broadly expressed from trachea to terminal bronchiole (arrowhead) in cells that expressed Scgb1a1 (B, D, F, H, arrows, brown = Scgb1a1 protein). *Cbr2* was expressed along the entire airway both, in cells that express Scgb1a1 (H, arrows) and others (H, asterisks). (**I–N**) Transcripts that were expressed in restricted patterns in the airways in cells that expressed Scgb1a1. *Hp* expression was negligible in the trachea (**I**, left inset) and mainstem bronchi (bracketed region shown at higher resolution in right inset) but high in the terminal bronchiole (**I,** arrowhead). *Hp* expressing cells in the intrapulmonary airways co-expressed Scgb1a1 (**J**, arrows). *Reg3g* expression detected in the trachea (**K**, left inset) and mainstem bronchi (bracketed region shown at higher resolution in right inset) but not in the terminal bronchiole (**K**, arrowhead). Many *Reg3g* expressing cells in the proximal airways co-localized with Scgb1a1 and a few cells did not (**L,** asterisk). Upk3a expressing cells were rare (**M**, arrow) and only detected in the vicinity of NEBs (Cgrp, red). (**N**) *Upk3a*-creER expressing cells were distributed throughout the intrapulmonary airways (arrowheads mark the bronchioalveolar duct junction). *Upk3a*-creER expressing cells in the adult lung (red, arrows) were clustered around NEBS (green) and expressed Scgb1a1 (white).

Among the eleven genes differentially expressed in E18.5 control and Rbpjk^CNULL^, at least three *(*Scgb1a1, Scgb3a2, Cyp2f2) have been well reported to label adult CCs [1], [6], [8]. We examined whether the other nine genes revealed by our approach were only transiently expressed in the E18.5 CCs or could recognize the mature CCs in the adult. To address this issue we performed double ISH-IHC in the sections of the adult lung and trachea and examined if cells expressing the transcript of interest also expressed Scgb1a1. Figure 3A–F shows the extensive overlap of *Scgb1a1, Scgb3a2 or Cyp2f2* mRNA with the Scgb1a1 IHC signals confirming that the cells expressing these transcripts are indeed CCs. Co-labeling with Scgb1a1 was also observed in the majority of cells expressing *Reg3g*, *Cbr2, and Hp*. The presence of cells single-labeled for *Cbr2,* and to a far lesser extent either *Hp* or *Reg3g* (Fig. 3H, L, asterisks), suggested that these genes are also expressed in other cell types of the adult lung. This was in agreement with the residual expression of *Cbr2* and *Hp* in the airway epithelium of E18.5 Rbpjk^CNULL^ (Fig. 2 J, L).

Expression of *Krt15*, *Chad, Lrrc26* and *Gabrp* in the adult lung, was undetectable by ISH compared to E18.5, suggesting that these genes may be expressed at higher levels in immature than mature CCs (not shown). *Upk3a*-expression was detected in rare cells associated with NEBs of the adult lung (Fig. 3M). Since NEBs are surrounded by CCs, the location of the Upk3a-expressing cells suggested that these cells are CCs and we expected them to express Scgb1a1. To investigate this we used an inducible *Upk3a*-creER knock-in transgenic strain to activate expression of reporter gene in *Upk3a-*expressing cells [12]. Double labeling for the reporter (Td-Tomato) and Scgb1a1 in the adult lung showed that the majority of the Td-Tomato^+^ cells were also Scgb1a1^+^ (Fig. 3N).

### CC Ablation Assay and Analysis of CC Reporter Mouse Confirm the Distribution of these Genes in Regionally Distinct CC Populations

**Figure 4:**
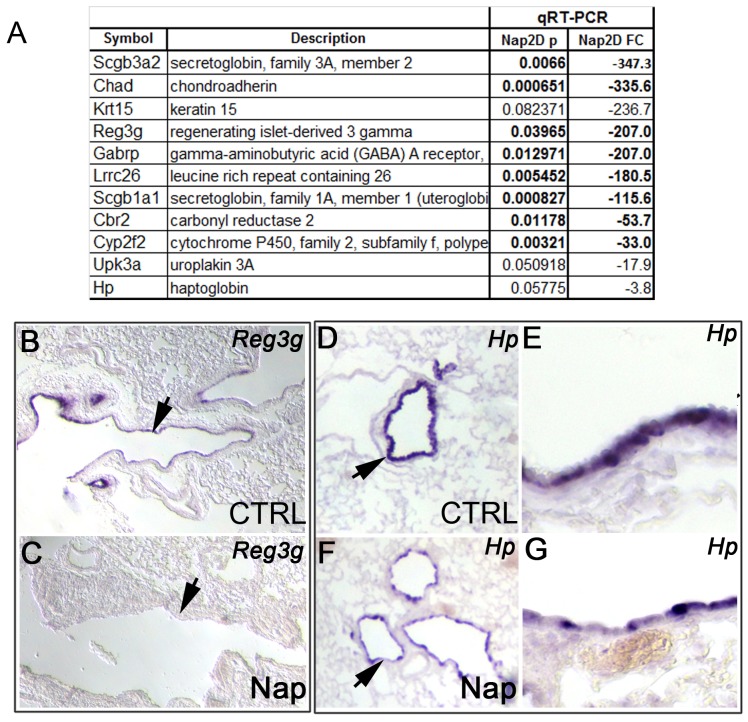
Impact of Naphthalene injury on the expression of genes identified by transcriptional profiling. **(A)** qRT-PCR analysis of gene expression in mock-treated (corn oil, CTRL) and Naphthalene-treated (Nap) lungs 2 day post-injection (n = 3 lungs per condition). (**B–G**) ISH for Reg3g (**B–C**) and Hp (**D–G** in CTRL (**B**, **D–E** and Nap-treated lungs (**F–G** respectively. **E**, **G** are higher magnification images of airways shown in **D**, **F** respectively.

Next, we reasoned that if the eleven genes identified in our screen are expressed in adult CCs, their expression would be profoundly altered when the integrity of CCs was compromised. To look into this issue we used a functional assay in which CCs were selectively ablated by Naphthalene (Nap) injury. Intraperitoneal administration of Nap in adult mice is known to lead to exfoliation of the vast majority of CCs as a function of their ability to selectively metabolize this cytotoxicant, resulting in cell death within the initial two days [14], [15]. To examine the expression of the candidate CC markers in Nap-treated lungs, adult mice were injected with corn oil (control) or Nap (300 mg/Kg body weight dissolved in corn oil), and total RNA was isolated from lung homogenates at 50–52 h (n = 3 lungs per condition). qRT-PCR analysis of the eleven genes in Nap-treated mice showed a significant decrease in expression relative to control for nearly all the genes studied (p<0.05, Fig. 4A). We noted that Reg3g expression was significantly downregulated post Nap (Fig. 4A, B–C) but Hp (p = 0.057) and UpK3a (p = 0.05) were not. We hypothesize that these genes are expressed in cells other than CCs post-Nap (Hp, Fig. 4D–G), or are expressed in CCs that escape Nap-dependent ablation (Upk3a). The location of Upk3a-expressing cells is consistent with this hypothesis. Three gene products not detected by ISH in the adult lung were found to be down-regulated post Nap based on qRT-PCR analysis (Chad, Gabrp, and Lrrc26). It is possible that these genes are expressed at low levels in the CCs in adults. Krt15 was the only gene that was both undetected in the adult airways (ISH) and unaffected by Nap.

**Figure 5:**
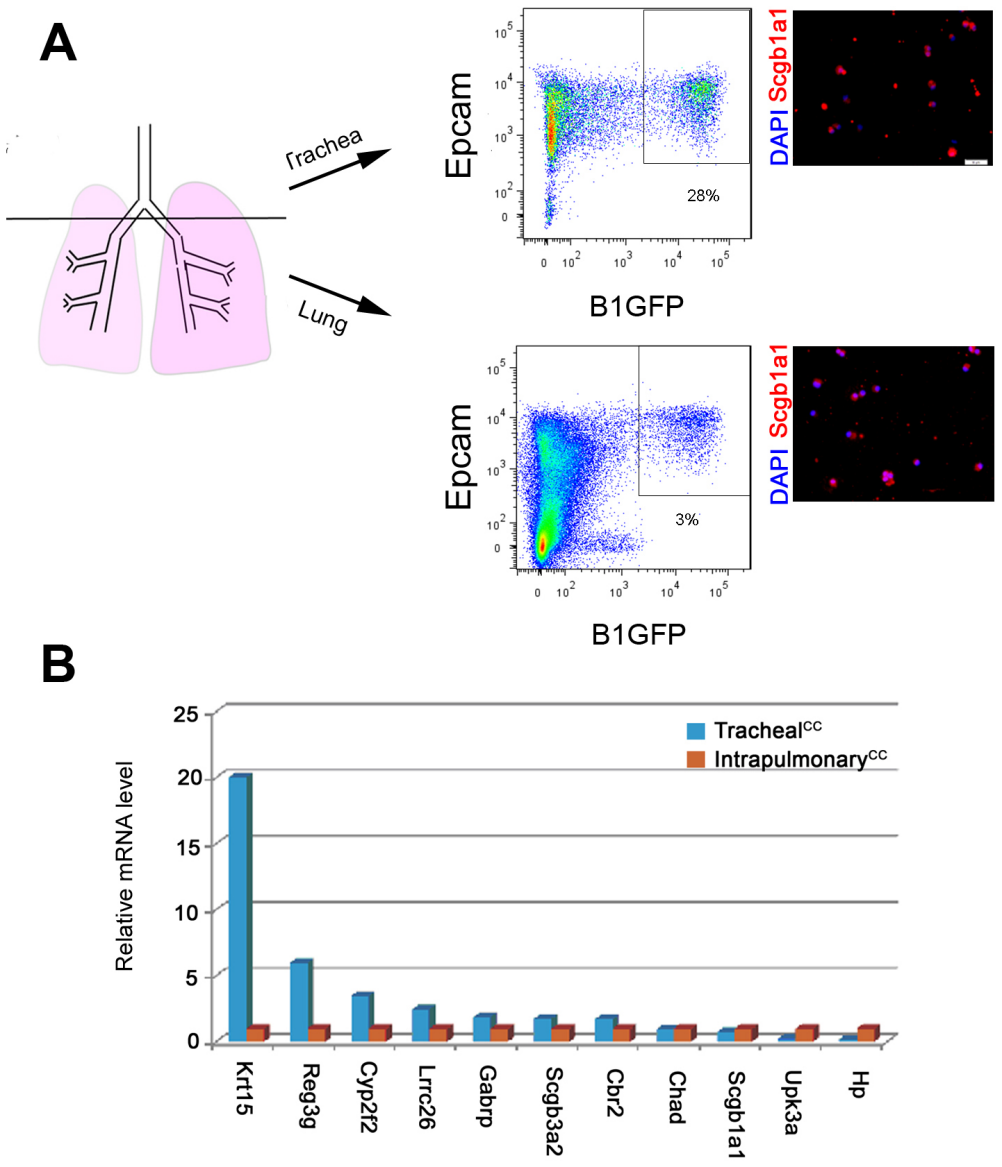
Comparison of gene expression in CCs derived from proximal (trachea) and distal (intrapulmonary) airways of B1-EGFP mice. **(A)** Isolation and characterization of B1-EGFP expressing (GFP^High^) epithelial (Epcam^+^) cells from the trachea and intrapulmonary airways. Dot plots (central panels) and IHC of cytospins (right panels) showing that the isolated cells from the respective pools expressed Scbg1a1. **(B)** qRT-PCR-based analysis of the relative expression of eleven genes in tracheal and intrapulmonary GFP^High^ Epcam^+^ cells.

Our ISH/IHC studies in the adult lung have shown that *Reg3g*, *Hp*, *Upk3a* expression is enriched in distinct CC subpopulations (Fig. 3). To corroborate these findings, we isolated and analyzed CCs from two distinct regions of the respiratory tract: trachea/extrapulmonary airways and intrapulmonary airways. For flow cytometry-based sorting of CCs from these regions we utilized adult B1-EGFP reporter mice. These mice carry an enhanced Green Fluorescent Protein (EGFP) transgene under the control of the promoter of the B1 subunit of the Vacuolar ATPase. The B1-EGFP mice express EGFP selectively in CCs and have been used for *in vivo* imaging and isolation of CCs [16]. Flow cytometry-based sorting followed by IHC analysis of cytospins showed that >90% and >80% of the cells from the respective pools expressed Scgb1a1 (Fig. 5A). IHC analysis also showed that ∼5% of the cells expressed the ciliated cell marker Foxj1 but none expressed the basal cell marker Trp63 (not shown). Based on this analysis we inferred that the sorted cell populations from trachea and intrapulmonary airways are highly enriched for CCs representative of these regions. qRT-PCR analysis of CCs confirmed that *Reg3g* was enriched in the extrapulmonary CCs, while *Hp* and *Upk3a* were enriched in the intrapulmonary CCs (Fig. 5B). This was consistent with the ISH studies in the adult lung.

We found that levels of *Krt15*, *Reg3g*, *Cyp2f2*, *Lrrc26*, *Gabrp*, *Scgb3a2*, and *Cbr2* were higher in tracheal and extrapulmonary CCs, while *Scgb1a1*, *Upk3a*, *Hp* were higher in the intrapulmonary CC population. This analysis also suggested that the expression characteristics of Chad and Krt15 were different in the embryonic and adult lung. *Chad* expression in the adult did not exhibit the proximal enrichment found in the embryonic lung. It is possible that pattern of *Chad* expression in the embryonic lung is not maintained in the adult lung or that the B1-EGFP transgene may not efficiently label a population of Chad-expressing CCs of the adult lung. The identity of the Krt15-expressing cells in the proximal airways will require further analysis.

### Stage-dependent Differences in Gene Expression during Early Airway Differentiation

**Figure 6:**
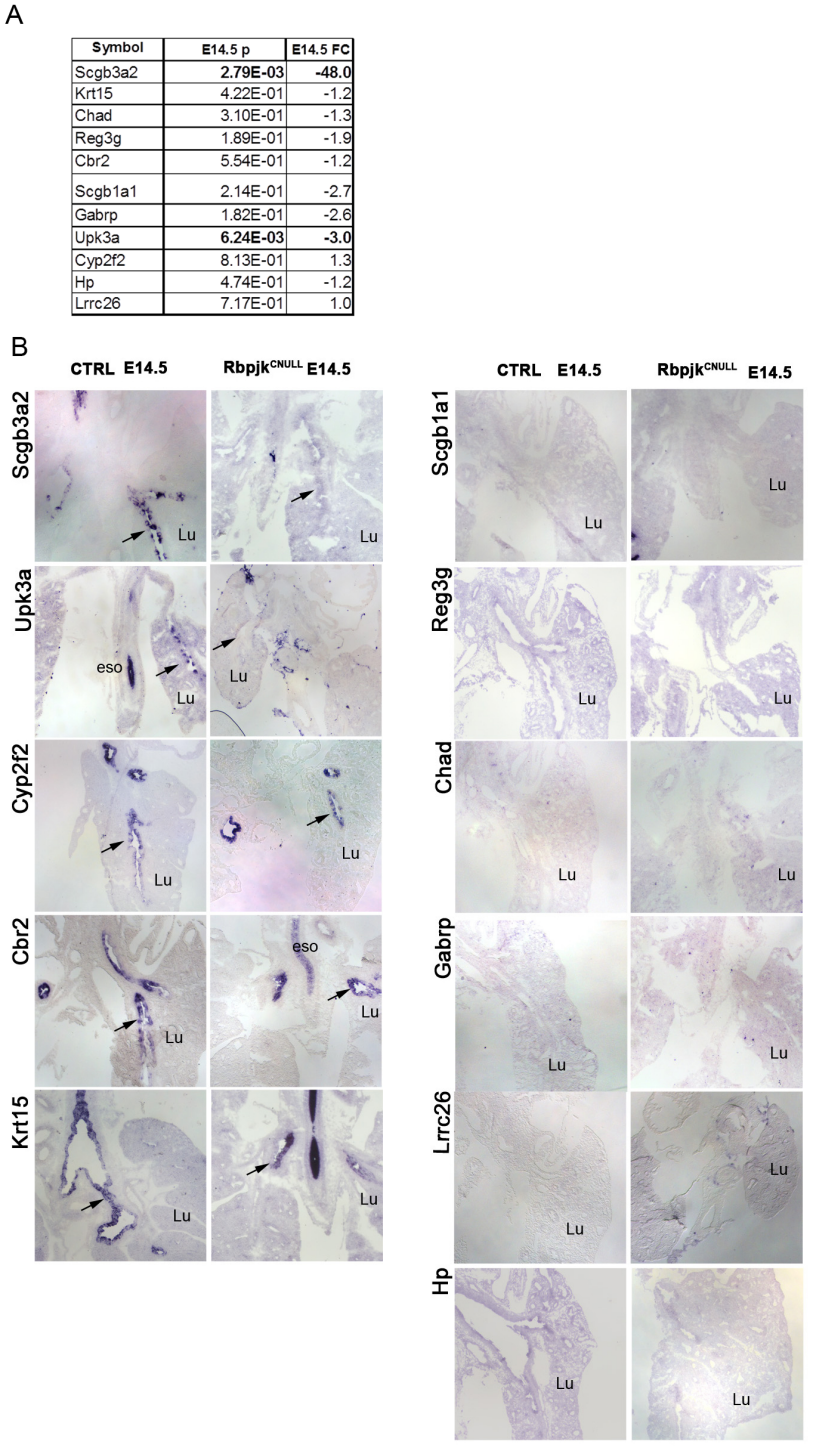
Expression patterns of the genes identified by transcriptional profiling at E14.5. **(A)** qRT-PCR analysis of gene expression in CTRL and Rbpjk^CNULL^ lungs from E14.5 **(B)** ISH showing expression of the eleven genes in lungs from control and Rbpjk^CNULL^ at E14.5. Genes expressed at E14.5 shown in left panel.

Next we asked whether the eleven genes selectively enriched in E18.5 CCs by our screen could recognize cells that are already committed to but not yet fully differentiated into the CC phenotype. It is still unclear when precisely the airways progenitors commit to the CC fate during lung development. At E14.5, prior to the onset of Scgb1a1 expression, at least two of the eleven genes found in our screen (*Scgb3a2, Upk3a*) have been described in the developing airway epithelium [9], [12]. This encouraged us to use E14.5 Rbpjk^CNULL^ and WT to investigate whether our genes could be potential markers of CC precursors at this stage. qRT-PCR analysis of E14.5 lungs confirmed that *Scgb3a2 and Upk3a* were indeed differentially downregulated in Rbpjk^CNULL^ lungs (Fig. 6A, p = 0.002 and p = 0.006 respectively). However, the expression the remaining nine genes were not downregulated in the mutants. To further investigate the pattern of expression of these genes at E14.5 we performed ISH in lung sections of control and Rbpjk^CNULL^ mutants. Interestingly, Cyp2f2, Cbr2, Krt15 could be detected unambiguously in the tracheal and proximal lung epithelium of E14.5 control and Rbpjk^CNULL^ lungs, suggesting that, at least at this stage, expression of these genes was not dependent on Notch signaling (Fig. 6B). Whether cells expressing these genes in Rbpjk^CNULL^ are precursors of secretory or non-secretory lineages is currently unclear. The distinct expression pattern of Scgb3a2 and Upk3a in E14.5 lungs suggests that the process of CC diversification occurs already at this stage in CCs precursors.

## Discussion

**Figure 7:**
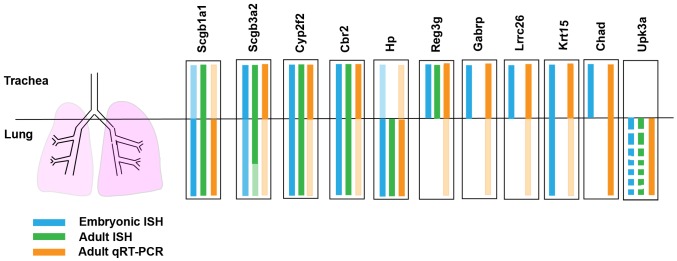
Summary of gene expression patterns in embryonic and adult lung. Cartoon summarizing the domains of expressions of the genes identified in Fig. 1 in the embryonic (E18.5) and adult lung based on ISH (blue, green) and qRT-PCR (orange) analysis.

In this study we have utilized a combination of gene array profiling, qRT-PCR and ISH-based analyses of genetic and injury models to identify candidate markers of CC precursors and CCs (summarized in Fig. 7). Profiling of Rbpjk^CNULL^, Notch signaling-deficient lungs in which CCs do not form, has identified several airway genes that are expressed in CC-precursors and CCs in the embryonic and adult lung. Most of these genes are downregulated in lungs in which CCs were ablated by Nap injury. Interestingly, several genes showed regionally restricted or localized patterns of expression that were similar in the developing and mature lung suggesting that they can be used to identify regionally distinct populations of CC throughout the P-D axis of the respiratory tract.

A transcriptional profiling study of CCs reported by Zemke and colleagues [17], a study that examined adult lungs post CC ablation, also identified *Scgb3a2*, *Scgb1a1*, *Cyp2f2*, *Gabrp* as CC markers but majority of the genes identified in this study did not overlap with ours (see Supplementary Fig. 1). There could be several reasons for this. Our microarray analysis differs from the above as it used a different type of microarray and was performed in the E18.5 lung instead of the mature and injured lung. Several genes identified by Zemke *et al* were not represented in our arrays (Supplementary Fig. 1). Conversely, genes such as *Upk3a* and *Chad* identified in this study were not represented in the arrays utilized by Zemke *et al* (B. Stripp, B. Brockway, personal communication). It has been reported that the onset of expression of several genes expressed in CCs occurs only after birth [13], [17].

There is accumulated evidence of major functional differences among CCs, depending on their P-D distribution or association with specific microenvironments in the lung [3], [4], [6], [18]. Our study supports the idea that the diversification of the CC phenotype already arises during embryonic development and identifies candidate markers that can be used to investigate this process. Regional differences in the CC phenotype are likely to arise from the combinatorial expression of multiple genes at different levels. We found that genes such as, *Scgb3a2*, *Cyp2f2, Cbr2* expressed throughout the airways (like *Scgb1a1),* do not facilitate distinction of proximal from distal CCs. By contrast, five of the eleven genes we identified here overlapped with Scgb1a1 but instead of having a Scgb1a1-like widespread expression pattern, showed revealing regional patterns. Transcripts of these genes were detected in proximal (*Reg3g*, *Chad*, *Gabrp*, *Lrrc26*), distal (*Hp*), and NEB-associated (*Upk3a*) subpopulations of CCs. Most of these genes have not been previously demonstrated to mark both the developing and the mature CCs and with distinct P-D patterns of transcript distribution.

Genetic studies have shown that the Notch signaling pathway is essential for the specification for CCs. Here we describe genes that are expressed from early stages but only dependent on Notch for their expression at late stages (*Cyp2f2*) and genes that are dependent on Notch but expressed in non-overlapping spatial domains (*Reg3g* vs. *Upk3a*). We have reported previously that disruption of Notch signaling in Rbpjk^CNULL^ mice results in a reduction in the expression of the transcription factor Sox2 throughout the airway epithelium at E18.5 (6). However, at E14.5, Sox2 expression in mutant animals is indistinguishable from controls (6). This time-dependent behavior is consistent with what we observed for *Cyp2f2*, *Cbr2*, *Hp*, and *Krt15*. The data suggest that other pathways must act in concert with Notch to regulate CC differentiation and diversification. It is also plausible that the distinct spatial patterns of Notch-dependent gene expression along the P-D axis are related to the different progenitors in distinct airway segments or microenvironments [19]. Future studies will explore this issue.

Mice expressing a constitutively activated form of Notch (NICD1) in the developing epithelium express Muc5AC and undergo mucus metaplasia. Interestingly, the competence to induce genes such as Muc5AC and undergo mucus metaplasia is acquired only by cells in the proximal airway epithelium, and that too, only later in development [11], [12] (A.G., W.V.C. unpublished observations). Several genes identified in this screen are enriched in the proximal airways (*Reg3g, Chad, Gabrp, and Lrrc26*) at late developmental stage. Whether the mechanisms that regulate the onset of expression of genes from proximal airway Clara precursors also regulate the competence for mucus metaplasia will be important to investigate.

## Materials and Methods

All animal work reported here has been conducted in accordance with the necessary guidelines approved by the Institutional Animal Use and Care Committee (IACUC) at Boston University. The IACUC at Boston University specifically approved this study. Any procedure that could conceivably cause distress to the animals employed peri-procedure anesthesia with isofluorane gas (Baxter Healthcare Corp.) delivered by an anesthetic vaporizing machine in our animal facility. In addition, all animals were monitored for signs of distress and euthanized if in distress. Euthanasia was performed by CO_2_ inhalation followed by cardiac puncture per guidelines at Boston University.

### Mouse Models

Rbpjk^CNULL^ lungs were generated by the airway-specific ablation of Rbp-jk using methods that have been described previously (6, 7). *Upk3a*-creERT2 and *Rosa26*-LSL-Td-Tomato mice were obtained commercially (Jackson Laboratory). For labeling Upk3a-expressing cells, Upk3a^cre/+^; Rosa^Td-tomato/+^ heterozygotes (6–8 weeks of age) were injected with Tamoxifen (Sigma, 0.25 mg/gm body weight) on three consecutive days to activate the Cre recombinase. Animals were sacrificed 1 week after the last injection s. Naphthalene injury experiments were performed using established protocols [17]. Briefly, FVB/n mice aged 8–12 weeks (Charles River) were injected intraperitoneally with corn oil (vehicle) or Naphthalene dissolved in corn oil (300 mg/Kg) between 10 am-12 pm and sacrificed at 50–52 hr. Mice that express enhanced Green Fluorescent Protein (EGFP) under the control of the promoter of the B1 subunit of the Vacuolar ATPase (B1-EGFP, B6CBAF1/J background) have been described previously [16].

### RNA Isolation

All lungs were dissected at or distal to the carina at all stages examined. For analysis of developmental stages, whole lungs were used for RNA isolation. For analysis of adult lungs in control and Naphthalene-treated animals, the left lobe was used for RNA isolation. Total RNA from lungs of various stages and genotypes was isolated using Trizol (Invitrogen) and reverse-transcribed using oligo-dT primers provided in the Superscript III kit (Invitrogen).

### Microarray Analysis

For microarray profiling, total RNA from E18.5 lungs was submitted for labeling and hybridization (Mouse Gene 1.0 ST Whole Genome Array, Affymetrix) to the Boston University Microarray Core facility. Raw Affymetrix CEL files were normalized to produce gene-level expression values using the implementation of the Robust Multiarray Average (RMA) in the Affymetrix package included within in the Bioconductor software suite (version 2.10.0) and an Entrez Gene-specific probe set mapping from BrainArray (version 14.0.0). Differential gene expression was assessed with Student’s two-sample *t* test, assuming equal variance, using the multtest package (see below). All microarray analyses were performed using the R environment for statistical computing (version 2.12.0). The microarray data is available in the Gene Expression Omnibus (GEO, Series ID GSE52926).

### qRT-PCR Analysis

The methods used for Quantitative Real Time PCR have been published previously [12]. Briefly, the following primers were obtained from Applied Biosystems: Scgb3a2 (Mm005044412_m1), Upk3a (Mm01301754_m1), Cyp2f2 (Mm00484087_m1), Cbr2 (Mm01246806_g1), Hp(Mm1239994_g1), Krt15(Mm00492972_m1), Scgb1a1(Mm00442046_m1), Reg3g(Mm01181783_g1), Gabrp(Mm01193034_m1), Lrrc26(Mm00525100_g1), Chad(Mm483284_m1), Pon1 (Mm00599936_m1), Fmo3 (Mm01306345_m1), Aox3 (Mm00508163_m1), Cldn10 (Mm01226326_g1) and β-actin (control). PCR reactions were constituted with the Assays-on-Demand kit (Applied Biosystems), and the samples were analyzed on an ABI 7000 instrument (Applied Biosystems).

### RNA in situ Hybridization, Immunohistochemistry, and Imaging

Embryonic lungs were fixed overnight in 4% (wt/vol) Paraformaldehyde in PBS (4°C) and embedded in OCT using established protocols. Adults lungs were inflated with 4% (wt/vol) Paraformaldehyde in PBS, immersed in the same fixative overnight (4°C) prior and then embedded in paraffin using established protocols. All fixatives were prepared fresh. Frozen sections of 8–10 µm thickness and paraffin sections of 5–6 µm thickness were utilized for ISH studies. For ISH on sections from adult lungs, the section were subject to antigen retrieval (high pH, Vector Laboratories) prior to the hybridization as this dramatically improved sensitivity [20]. The T7-linked (antisense) and T3-linked (sense, control) gene-specific primers were utilized for PCR and riboprobe syntheses are listed in Table 1. The protocols for probe synthesis have been described previously (9). For double ISH/IHC, sections were processed for IHC directly after ISH. Goat anti-Scgb1a1 (Santa Cruz), Mouse anti-RFP (Abcam) and Rabbit anti-Cgrp (Sigma) were used to label CCs, Td-tomato-expressing and neuroepithelial cells respectively. All samples were imaged on a Nikon Labophot-2 microscope equipped with a Nikon Digital Sight DS-Ri1 CCD-camera or on a Zeiss LSM-710 metaconfocal laser-scanning microscope.

**Table 1:** 

	Forward Primer	Reverse Primer
**Scgb1a1**	5′AATTAACCCTCACTAAAGGGAGGTCCTGGGAGCATCTTCT 3′	5′TAATACGACTCACTATAGGGTCAACCGAACATTGTCAGGA 3′
**Scgb3a2**	5′AATTAACCCTCACTAAAGGGAGGTGACAGCGAGCAGAACT 3′	5′TAATACGACTCACTATAGGGCACGTAGCAAAGGCTTCTCC 3′
**Cyp2f2**	5′AATTAACCCTCACTAAAGGGGGAACTTTGGAGGCATGAAA 3′	5′TAATACGACTCACTATAGGGAACTCCTGAGGCGTCTTGAA 3′
**Cbr2**	5′AATTAACCCTCACTAAAGGGTATCGGCCCTGTGGACTTAC3′	5′TAATACGACTCACTATAGGGCAACCTCTGCGAACTTCCTC3′
**Hp**	5′AATTAACCCTCACTAAAGGGTCTACGTGGGGAAAAACCAG3′	5′TAATACGACTCACTATAGGGCATGTCATGAATGGCAAAGG3′
**Krt15**	5′AATTAACCCTCACTAAAGGGTGGAGATGCAGATTGAGCAG3′	5′TAATACGACTCACTATAGGGGGTAATGACCCCCTGGATCT3′
**Reg3g**	5′AATTAACCCTCACTAAAGGGTCAGGTGCAAGGTGAAGTTG3′	5′TAATACGACTCACTATAGGGGGCCTTGAATTTGCAGACAT3′
**Chad**	5′AATTAACCCTCACTAAAGGGACCACACTGAAACACGTCCA3′	5′TAATACGACTCACTATAGGGGGAGCTGGGATGGTGATAGA3′
**Gabrp**	5′AATTAACCCTCACTAAAGGGCTCCCTCGATTCAGTTCCTG3′	5′TAATACGACTCACTATAGGGATTGCTGGGGTTTTGAATTG3′
**Lrrc26**	5′AATTAACCCTCACTAAAGGGAGCCTGCAGGACAATTCACT3′	5′TAATACGACTCACTATAGGGCGGGGTCTAGCTGTCTCCTT3′
**Upk3a**	5′AATTAACCCTCACTAAAGGGGTGGCTGGACTGTGAACCTC 3′	5′TAATACGACTCACTATAGGGTTGCCCACCCTGACTAGGTA 3′

### Flow Cytometry and Cell Sorting

Tracheas and lungs from B1-GFP mice were separated, cut into pieces and then incubated in papain dissociation solution according to the manual (LK003153, Worthington Biochemical Corporation). Dissociated cells were then passed through a 40mm cell strainer in PBS containing 2% FBS. DAPI as a viability dye was added to a final concentration of 200ng/ml before sorting. Anti-mouse Epcam (eBioscience) was used to sort out specifically epithelial cells. GFP sorting was performed on FACS Aria and data was analyzed with FACS Diva (BD Biosciences). Cells were sorted directly into Trizol for RNA extraction.

## Supporting Information

Figure S1 **Comparison of genes identified in this study with genes identified by transcriptional profiling of adult lungs post CC ablation.** (**A**) Venn diagram showing genes downregulated in Rbpjk^CNULL^ lungs at E14.5 (qRT-PCR), E18.5 (microarray, qRT-PCR) and genes downregulated in the adult lung post Naphthalene (Nap) and Ganciclovir (CCSP HSV tk) mediated CC ablation, see text, [17]). Scgb3a2 (in grey) is the gene that most consistently recognizes the CC phenotype in lungs from E14.5 to adulthood. In addition to Scgb3a2, three other genes found at E18.5 overlap with those reported by profiling of the adult injured lung (purple).
(TIF)
