# Supplementary figures and images for: Analysis of Notch Signaling-Dependent Gene Expression in Developing Airways Reveals Diversity of Clara Cells
Source: PLoS One. 2014 Feb 21;9(2):e88848. doi: 10.1371/journal.pone.0088848 (PMC3931645; doi:10.1371/journal.pone.0088848)

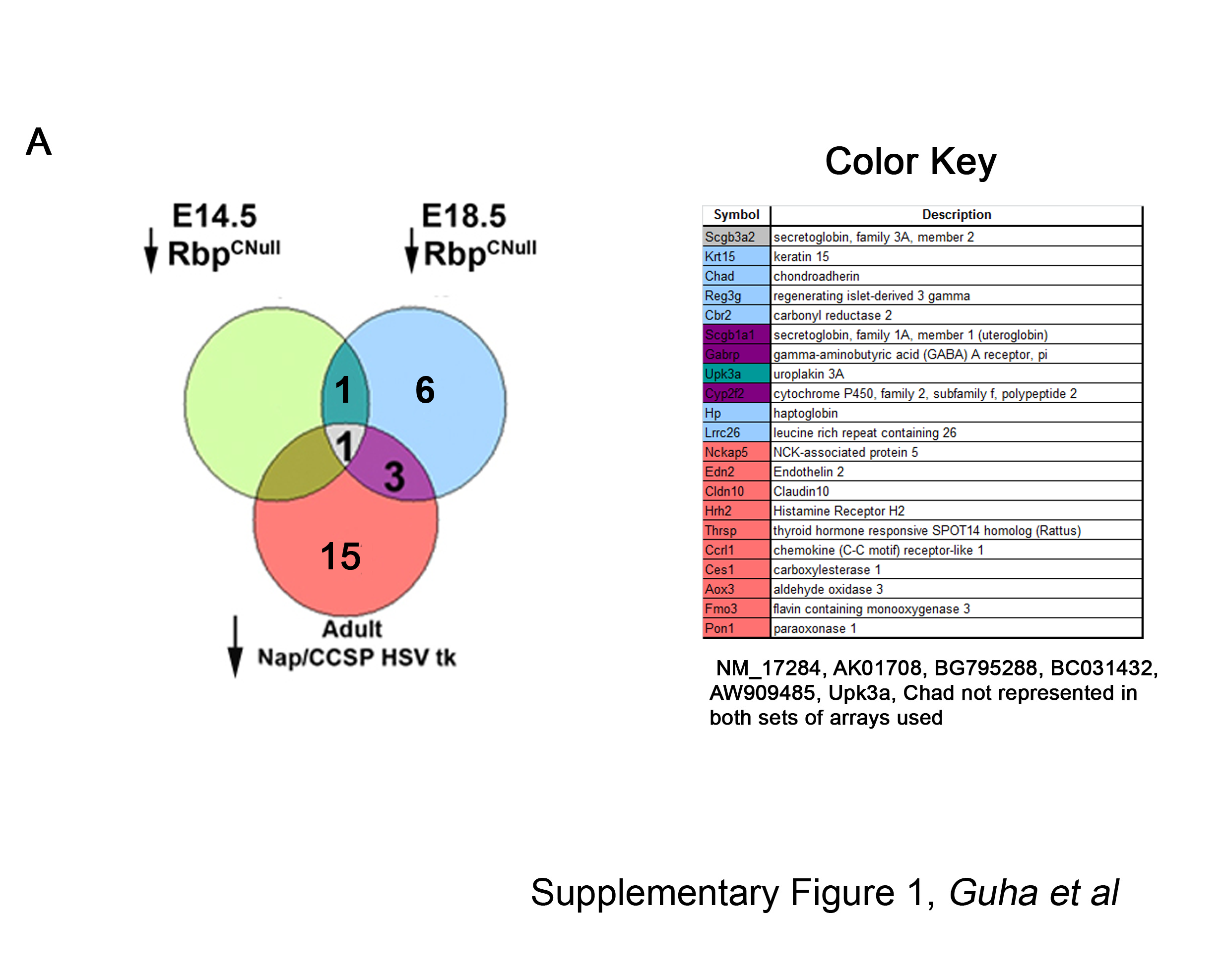

Supplement: Figure S1 — Comparison of genes identified in this study with genes identified by transcriptional profiling of adult lungs post CC ablation. (A) Venn diagram showing genes downregulated in RbpjkCNULL lungs at E14.5 (qRT-PCR), E18.5 (microarray, qRT-PCR) and genes downregulated in the adult lung post Naphthalene (Nap) and Ganciclovir (CCSP HSV tk) mediated CC ablation, see text, [17]). Scgb3a2 (in grey) is the gene that most consistently recognizes the CC phenotype in lungs from E14.5 to adulthood. In addition to Scgb3a2, three other genes found at E18.5 overlap with those reported by profiling of the adult injured lung (purple). (TIF) [file pone.0088848.s001.tif]
